# Insights From Flutracking: Thirteen Tips to Growing a Web-Based Participatory Surveillance System

**DOI:** 10.2196/publichealth.7333

**Published:** 2017-08-17

**Authors:** Craig Dalton, Sandra Carlson, Michelle Butler, Daniel Cassano, Stephen Clarke, John Fejsa, David Durrheim

**Affiliations:** ^1^ HMRI University of Newcastle New Lambton Heights Australia; ^2^ Hunter New England Population Health Hunter New England Local Health District Wallsend Australia; ^3^ Chord Wizard Systems Mayfield Australia

**Keywords:** epidemiology, surveillance, influenza, user centered design, World Wide Web

## Abstract

Flutracking is a weekly Web-based survey of influenza-like illness (ILI) in Australia that has grown from 400 participants in 2006 to over 26,000 participants every week in 2016. Flutracking monitors both the transmission and severity of ILI across Australia by documenting symptoms (cough, fever, and sore throat), time off work or normal duties, influenza vaccination status, laboratory testing for influenza, and health seeking behavior. Recruitment of Flutrackers commenced via health department and other organizational email systems, and then gradually incorporated social media promotion and invitations from existing Flutrackers to friends to enhance participation. Invitations from existing participants typically contribute to over 1000 new participants each year. The Flutracking survey link was emailed every Monday morning in winter and took less than 10 seconds to complete. To reduce the burden on respondents, we collected only a minimal amount of demographic and weekly data. Additionally, to optimize users’ experiences, we maintained a strong focus on “obvious design” and repeated usability testing of naïve and current participants of the survey. In this paper, we share these and other insights on recruitment methods and user experience principles that have enabled Flutracking to become one of the largest online participatory surveillance systems in the world. There is still much that could be enhanced in Flutracking; however, we believe these principles could benefit others developing similar online surveillance systems.

## Introduction

**Figure 1:**
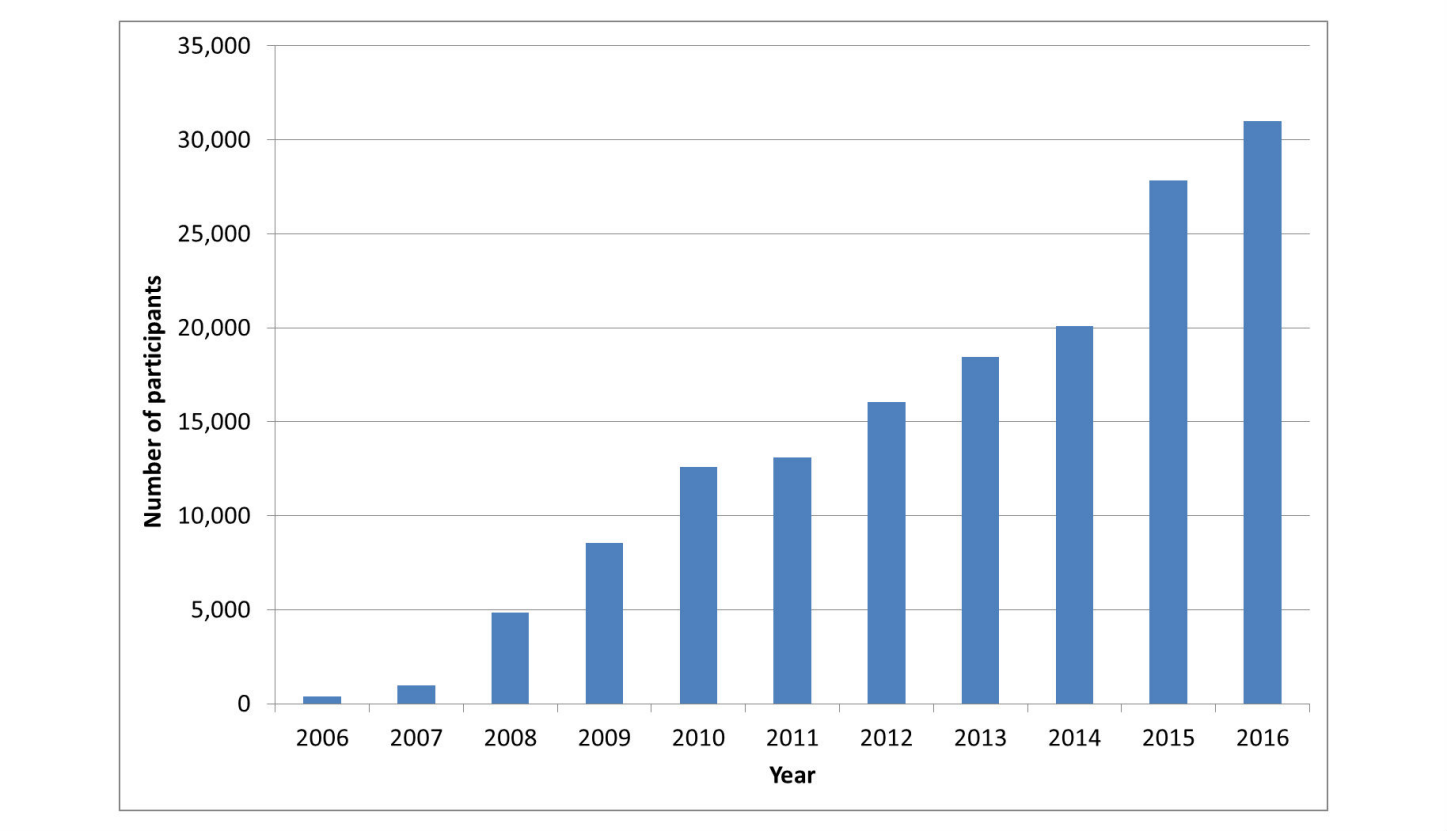
Number of participants completing at least one survey by year, 2006 to 2016, Flutracking, Australia.

Flutracking (www.flutracking.net) is a weekly Web-based survey of influenza-like illness (ILI) in Australia that has grown from 400 participants in 2006 to over 26,000 participants every week in 2016, with 30,900 participants completing at least one survey (Figure 1). Flutracking monitors the transmission and severity of ILI across Australia [1,2]. The survey documents symptoms (cough, fever, and sore throat), time off work or normal duties, influenza vaccination status, laboratory testing for influenza, and health seeking behavior. The project was inspired by the publication of an article in 2005, “‘Did you have the flu last week?’ A telephone survey to estimate a point prevalence of influenza in the Swedish population” [3]. We believed that an online platform would allow a larger, less expensive, and continuous assessment of ILI incidence and morbidity.

Community based surveys of ILI, such as Flutracking, are integral to comprehensive influenza surveillance which also incorporates complementary primary care, emergency department, hospital, intensive care unit (ICU), mortality, and laboratory surveillance. They can provide a unique insight into influenza epidemiology as they are not distorted by jurisdictional practices, health seeking, or practitioner behavior. Early in the 2009 H1N1 influenza pandemic, Flutracking was able to identify that the community level ILI attack rates were not appreciably different than they had been in the past seasonal influenza years. Rather, the high rates of ILI reported from emergency departments and influenza laboratory confirmations were due to increased health-seeking behavior and increased laboratory testing, respectively, due to the pandemic [4].

In this paper, we share our learnings on recruitment and retention of participants that have enabled Flutracking to become one of the largest online participatory surveillance systems in the world. In listing our recommendations, we divide the paper into two parts: (1) recruiting participants and (2) retaining participants.

### Recruiting Participants: Lessons Learned

We initially sent an invitation email to approximately 7000 employees of the Hunter New England Area Health Service in South-Eastern Australia. Additionally, an invitation to join was included in the Health Service newsletter. These two efforts jointly resulted in 400 participants completing at least one survey in 2006. We have identified seven lessons learned from our recruiting efforts so far.

#### Ask Participants to Invite Their Friends to Join

Since 2012, the current participants were emailed a week or two prior to the commencement of the survey each year and were asked to invite their friends or colleagues to join. This became one of our main recruitment methods, with typically over 1000 new participants in the week following the annual appeal to participants. We split tested emails and found that asking participants to invite a concrete number of people to join (eg, 2 or 3) is more successful than a generic “invite your friends” request. Comparing 1000 emails sent to participants who were asked to invite “friends” with 1000 emails sent asking to invite “3 friends,” we found that the former resulted in 79 new participants versus 182 for the latter.

#### Leverage Organizational Email Invitations

After the 2006 pilot, we expanded Flutracking beyond the Hunter New England Area Health Service and accepted participants from across the state in 2007 and then nationally. From 2008 to 2013, we sought to identify organizations that would be willing to disseminate the invitation to join Flutracking via their corporate email networks. We reviewed lists of Australian companies and government organizations with high numbers of employees and then selected those with two features: (1) a high proportion of staff sitting in front of computers with Internet access and frequent email use and (2) an assumed high level of staff autonomy (eg, not a “call center”).

We telephoned selected organizations and asked to speak to the “head of occupational health and safety” or, secondarily, “the director of human resources,” assuming this position would be most amenable to discussing an intervention that could raise awareness about respiratory illness in the workplace. We telephoned about 500 organizations each year prior to the survey commencement, with approximately 30 organizations agreeing to circulate an invitation email to their workforce each year.

Health departments were asked to assist with recruitment; they were also offered jurisdictional specific data once a threshold of 1000 participants was reached. An email containing a clickable link to Tasmanian public health staff in 2008 recruited 556 participants in 2 days and by the end of the 2008 season, through continued promotion, Tasmania had recruited 1235 respondents. A newsletter with a clickable link promoting enrolment in Flutracking was sent to all South Australian Health employees on May 25, 2010. Flutracking participation in South Australia increased from 197 to 2189 participants for the week ending on May 30, 2010.

#### Seek Champions

The Director of Public Health in Tasmania, the southernmost state of Australia, was a strong advocate for Flutracking. The Director sent a personally signed memo encouraging participation in Flutracking via the Tasmanian Department of Health and Human Services email network and promoted enrolment through internal online newsletters. In 2015, Tasmania’s peak year of participation, approximately one of every 250 Tasmanians (population of 517,000) were participants. Repeated annual promotions by the health department have maintained Tasmania as the highest participating jurisdiction in Australia.

#### Prioritize Electronic Mass Media With Clickable Hyperlinks

Media releases promoting Flutracking were issued each year in mid to late April before commencing the first survey in May. There has always been good coverage by both print and online newspapers, radio, and television at both the regional and national levels. It is very clear, however, that the driver of recruitment following media coverage is not necessarily the audience of a media outlet, but rather the immediacy and longevity of clickable hyperlinks to our flutracking.net website. An online Australian newspaper that featured an interactive map of our data led to over 4000 referrals to our website in 2016. Referrals from this site were associated with peaks in joining of up to 100 new participants in a single day early in the year. Following radio and television promotion of Flutracking, we monitored postcode regions within the estimated audience zone for impacts on recruitment. For example, a radio interview with a typical audience of approximately 10,000 resulted in only four people joining in the hour following a direct appeal for listeners to join during the broadcast. An unusually successful radio interview with a typical listener audience of 300,000 led to approximately 230 more participants enrolling in the 24 hours following the broadcast; however, this radio program also featured a link to Flutracking on their website. Radio and TV promotions require the listeners to recall and enter a website address in order to join. This contrasts with the immediate response to clickable links in online articles and in the email and online newsletter promotions in the South Australian and Tasmanian health departments described above. In the days following a media release, we conducted Google News searches for mention of “Flutracking” to identify online news coverage that lack hyperlinks to the flutracking.net website and request that the survey site be hyperlinked.

#### Use Social Media and Website Analytics

We have used Facebook to promote Flutracking since 2011. Most posts are “boosted” by paying a fee to increase its audience reach, as this is a relatively minor cost compared with the time involved in planning and formatting a post. Facebook is useful for surfacing frequently asked questions from participants (eg, whether participants should answer the survey when travelling), educating participants on the differences between ILI and influenza, sharing surveillance insights, and directing potential participants to the website to enroll.

We use Google Analytics to analyze the number of referrals to the Flutracking join page from Facebook as well as from other promotional sites. Approximately 300 participants join each year after referral from our Facebook page. We used a unique, short URL tracking link for each promotional strategy so that we can split test different subject headings and messages in invitation emails to determine which combinations generate the most new joins.

#### Invite Participants to Report on Their Household Members

In 2008, we invited current participants to begin answering for their immediate household members. This resulted in a 44.89% (1163/2591) increase in the number of participants on average per week in 2008. Household participants continued to be an important component of our survey base with 39% of participants being a household member of a primary respondent in 2016.

#### Do Not Use Barriers Such as Usernames and Passwords

Passwords and usernames are barriers to participation in any online system [5]. Instead of using passwords, we sent a unique link to each individual user for each specific week so that regardless of the order in which a user responds to the survey (eg, some participants answered surveys in reverse chronological order after missing some survey weeks), the data are captured for the appropriate participant-weeks. The link was cryptically encoded to prevent malicious interventions from generating valid links and entering false data into the database and to prevent users from accessing other users’ data.

### Retaining Participants: Lessons Learned

Although total recruitment is an important parameter for success, long term week to week participation is an equally important determinant of data quality. Continued year to year participation supports cohort analyses, with retention of cohorts over multiple years allowing for within-person comparisons (eg, comparisons between the ILI experiences of individuals in years that they were vaccinated against influenza versus when they were not and changes in vaccination uptake following incidents such as the adverse pediatric reactions to a pandemic vaccine in 2010) [6].

**Figure 2:**
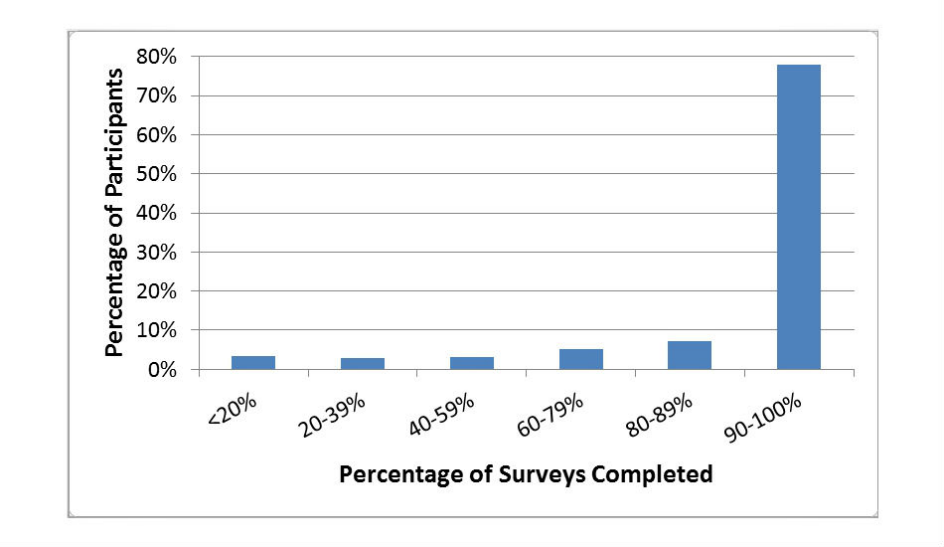
Percentage of participants who completed <20% to 90-100% of the 26 Flutracking surveys conducted in 2015.

**Figure 3:**
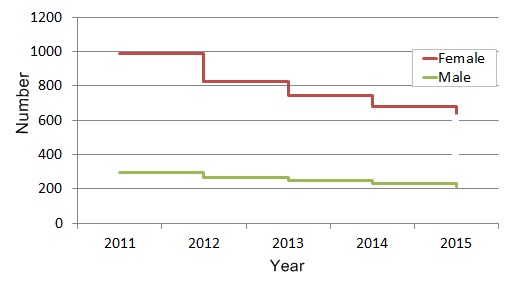
Five-year participation survival curve of 1400 survey respondents who joined in 2011 based on gender.

In 2015, 78% of participants who completed at least one survey in the first month of surveillance completed 90% or more of all 26 surveys that year (Figure 2). Although there has been a gradual loss of some participants each year, more than 60% of participants who joined in 2011 maintained participation over a 5-year period (Figure 3).

#### Consider the Short-Term Versus Long-Term Value of Inducements/Rewards for Participation

Flutracking has never offered rewards or inducements for participation. However, another online surveillance network, FluNearYou, has used significant recruitment inducements including iPads and rewards of US $10,000 for individuals and US $25,000 for groups reaching target recruitment goals [7]. Whereas inducements may increase initial recruitment, it is unclear whether they lead to more consistent participation. A trial of the impact of incentives on participation in online symptom surveys in Japan revealed that response rates were higher among the intervention arms with lower payments [8]. We conducted open-ended interviews with 30 participants who had participated in Flutracking for 6 years and asked, “Why have you participated in Flutracking for so many years?” Paraphrasing participant responses, they stated that the survey was quick and that they felt they were doing something useful for health research on a Monday morning. It is possible that short term inducements may only induce short term participation and that the best inducement is the opportunity to contribute to health surveillance and research.

Most surveillance practitioners believe it is important to provide feedback to participants [9]. Flutracking participants receive a link to a map and weekly report on influenza activity after they submit their weekly survey. While it is believed that rapid reporting of results is important to maintain support for a surveillance system, in August 2015, 116,773 surveys were completed, but there were only 2670 unique page views of Flutracking maps (Flutracking participants and non-participants combined) and 454 unique views of our online weekly report by the 28,500 unique users of the site. This suggests that less than 10% of website users who visit flutracking.net engage with the reports in the month of peak influenza activity.

#### Collect the Absolute Minimum Dataset (to Make the Survey as Quick and Easy as Possible)

Reducing the number of questions on recruitment and in the weekly survey decreases the burden on participants. Thus, Flutracking focuses on collecting the minimum dataset required to fulfill present day surveillance objectives. We avoided collecting data on any variable for which we did not have an immediate plan for analysis. On recruitment we only collected month and year of birth, sex, identification as an Aboriginal or Torres Strait Islander, highest educational attainment, postcode of residence, whether each participant works face to face with patients, and receipt of the previous years’ influenza vaccine. For example, having no initial analysis objective using the sex of participants we did not start collecting the sex of Flutracking participants until 2012. Initial analysis of our four years of collecting these data indicate a high female to male ratio of our participants and some divergence between males and females in participation patterns, with males exhibiting a greater retention rate year to year from 2011 to 2015 (Figure 3). Similarly, we did not collect any underlying medical condition. Granted such a condition might be a confounder or modifier of immunity, vaccination status, or health-seeking behavior, we will not add this variable until we are certain of its usefulness to inform an important surveillance objective.

**Figure 4:**
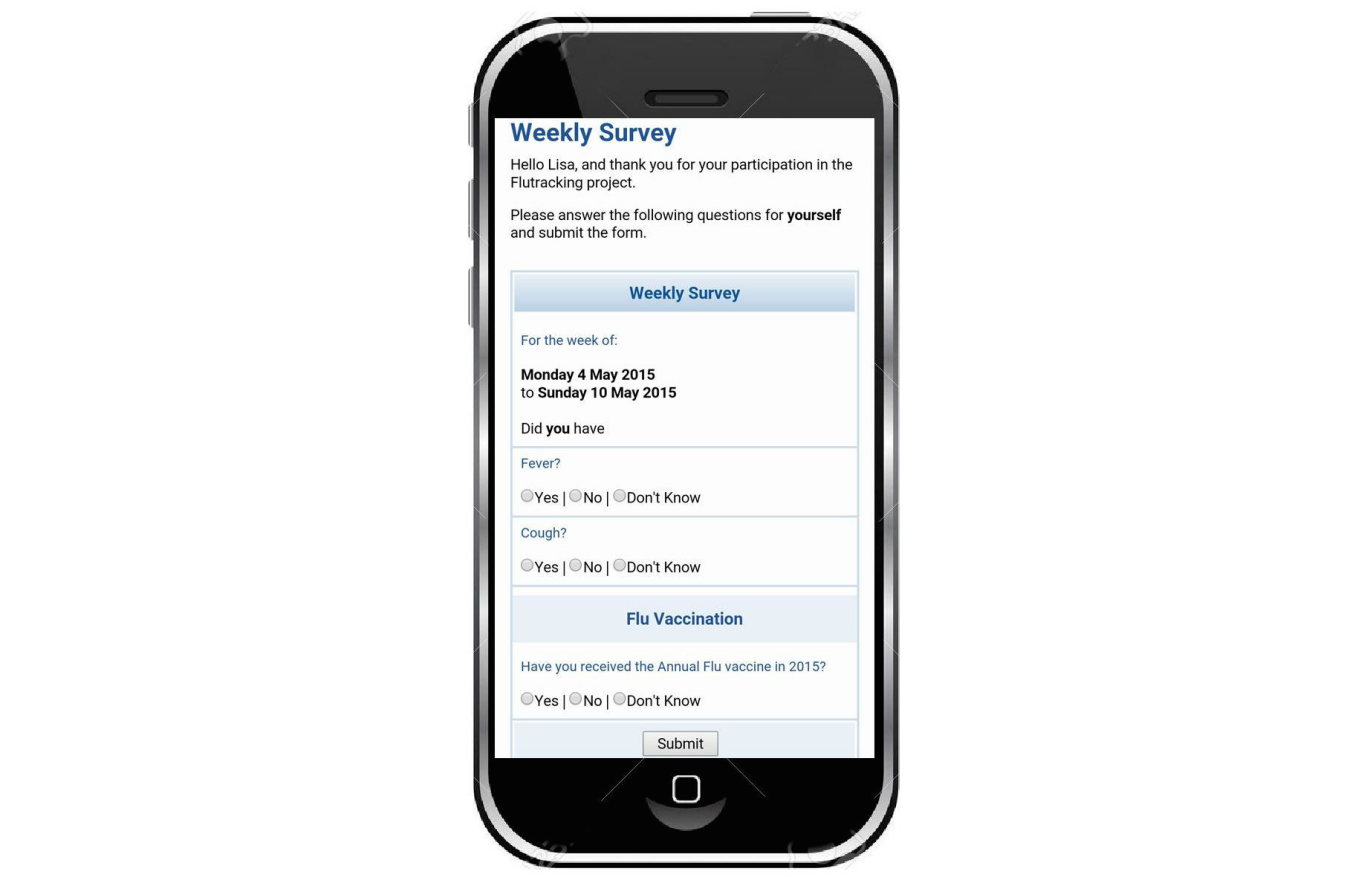
Screenshot of first screen of the flutracking.net survey, 2016.

We collected a minimal symptom profile comprising fever, cough, sore throat (if “yes” to both fever and cough), health care sought, days absent from normal duties, and collection and result of laboratory testing. We have been encouraged to adopt expanded symptom profiles that would allow comparison with established national and international case definitions for influenza, but resisted this move so far. The weekly survey displayed only three questions (Figure 4).

Specifically, participants were asked about any cough, fever, or vaccination in the last week (unless the participant had reported being immunized earlier in the year). If the respondent clicked no to either cough or fever, no more questions were presented and the survey was complete; this took less than 5 seconds for a survey on a single person. If the respondent clicked yes to both cough and fever, they were asked about sore throat and then time off work or normal duties and health-seeking behavior. Although asking about more symptoms (ie, more than three) and eliciting a measured temperature could enhance the predictive value of the collected data [10-12], we were not convinced that a minimal increase in predictive value justified extending the survey length.

Asking more questions about symptoms makes for a longer survey, which can result in reduced participation; we and the other designers of online surveillance systems need to strike a balance between tracking multiple syndromes (eg, respiratory, gastrointestinal, and neurological) versus a single syndrome. A screening “yes or no” question about the presence of a collection of symptoms can keep a multiple syndrome survey shorter than it would otherwise be. We are currently split testing this method to determine whether it has any impact on participation or data quality.

#### Segment Participants Based on Their Preferences for Survey Duration and Length

There is a long history of audience segmentation in public health practice [13]. We segmented our participants one time based on the preference to continue surveys over the southern hemisphere summer of 2009-10. Flutracking did not routinely continue surveillance over the summer period, which means we were unable to detect out-of-season ILI activity. We made this decision because participation often decreased toward the end of winter. When the weather warmed up, we sometimes received emails from participants with “Are we done yet?” undertones. We made an exception following the 2009 H1N1 pandemic and continued surveillance through the summer by asking respondents to opt in to continued surveillance past the usual mid-October end date. In total, 80.89% (5541/6850) of participants chose to continue to participate. The 2009 pandemic was an exceptional event, and we did not expect that 80% of the cohort would opt-in to continued year round surveillance. However, we believe that given the broad range of motivations and preferences for survey content and length, any further expansion of question number or content should be explored by allowing respondents to opt-in. Although this may produce a self-selection bias, we believe that it is inevitable in this type of surveillance and has to be balanced against participant preferences and participation.

#### Conduct Usability Testing to Optimize User Experience

We learnt while designing the Flutracking survey interface that we inevitably built our own assumptions into the design and were unable to assess it as a naïve user would. Inviting a naïve user to test the interface was critical (eg, to test whether radio buttons should be placed before or after potential answers and whether vertical lines between answers assist users to click on the intended radio button). Usability testing ranges from creating simple paper based mock-ups of draft screens using a word processing or publishing package to testing an operational online module. There are extensive guidelines on how to conduct usability testing, but the basic approach that we use is outlined in Textbox 1 [14].

**Basic usability testing conducted routinely by Flutracking (with paper- or screen-based systems).**

- Show the template to the user for two seconds and then conceal it. Ask: “What do you think this page is for, what can you do on it?” “What would you do on it?”
- Show the template again and ask: “Tell me again what you think this is for.” “What is your eye drawn to?” “Is there anything that looks confusing or surprising?” “What do you think the designer of the website wants you to do on this page?” ”What do you feel like clicking on?” “What do you think will happen if you click on that?” The test supervisor notes where the user’s expectations differ from what actually happens.
- This testing can also be conducted using “concurrent talking aloud” in which the naïve tester is asked to provide a “stream of consciousness” commentary in which they articulate every thought about the web template including what their eye is drawn to, what they are thinking about clicking on, what they expect to happen, and whether they are surprised or confused about what happens.
- Provide the tester with a “sickness scenario” and ask them to complete the illness questions.
- Ask the test user “what sort of illness would someone have who answered ‘yes’ to this question?”

#### Try to Adopt “Obvious Design” Principles (Again, to Optimize User Experience)

We tried to adopt “obvious design,” that is, there should be minimal need for explanations or textual guides to how the survey operates. Design should dictate flow without placing a cognitive load on the user. The Flutracking team is alert that any inclination to place a text explanation in our forms (eg, “click here to...” or “scroll down to...”) indicates a design flaw where user action is not obvious. To assist with obvious design, we tried to incorporate design elements that will be familiar to Internet users, for example, by copying terms, user flow, colors, and button design elements from popular online platforms such as Google or Facebook.

#### Technology Platforms: Web Survey, App, SMS, or Email?

Technology platform options such as email versus SMS triggered notifications of Web-based versus mobile phone app-based surveys might well impact upon recruitment or retention. We have only used emails and online surveys whereas FluNearYou has additionally used a mobile phone app. We found that Vaxtracker, a vaccine adverse event surveillance system, achieved higher participation among parents who signed up to receive both email and SMS reminders for surveys. We noted the emerging trend against native and hybrid apps because of compatibility issues and the need to recode both Web and mobile app platforms for any system or survey upgrades. The Gov.UK Digital Services Manual strongly discourages use of apps recommending the use of responsive mobile websites and emerging progressive app technology which capture the benefits of both responsive Web and native apps [15]. We recommend the use of responsive web design with future consideration of progressive Web applications with notifications to alert participants to new surveys.

## Discussion

The flutracking.net has grown rapidly over 10 years of its operation and maintained high participation rates. Invitations from existing participants to friends and colleagues remain the most successful recruitment method. Minimizing the length of the survey and ensuring design simplicity has been an absolute commitment that we believe contributed to our high participation rates. In addition to the insights offered here, user feedback and error detection have revealed the importance of prompting completion of missed surveys, timing follow-up of influenza laboratory results, and developing an administrative platform that runs weekly error checks to detect system and email gateway errors. These will be explored further in future publications. While Flutracking shares some common features with other online influenza surveillance networks such as FluNearYou and Influenzanet, their unique features provide opportunities for comparison [16,17]. We welcome dialogue and collaboration with other groups exploring online surveillance systems.

## Abbreviations

ILI: Influenza-like illness
